# PGRMC1 regulation by phosphorylation: potential new insights in controlling biological activity!

**DOI:** 10.18632/oncotarget.10691

**Published:** 2016-07-19

**Authors:** Michael A. Cahill, Jalal A. Jazayeri, Zaklina Kovacevic, Des R. Richardson

**Affiliations:** ^1^ School of Biomedical Sciences, Charles Sturt University, Wagga Wagga, NSW, Australia; ^2^ Molecular Pharmacology and Pathology Program, Department of Pathology, Bosch Institute, University of Sydney, Sydney, NSW, Australia

**Keywords:** signaling, phosphorylation, cytochrome P450, cancer, SH2-domain

## Abstract

Progesterone receptor membrane component 1 (PGRMC1) is a multifunctional protein implicated in multiple pathologies, including cancer and Alzheimer's disease. The recently published structure of PGRMC1 revealed heme-mediated dimerization that directed the PGRMC1-dependent cytochrome P450-mediated detoxification of doxorubicin. We describe here how the PGRMC1 structure also enables important new insights into the possible regulation of PGRMC1 function by phosphorylation. Predicted regulatory interaction sites for SH2- and SH3-domain proteins are in non-structured regions that could be available to cytoplasmic enzymes. Further to the published interpretation, we suggest that phosphorylation of PGRMC1 at position Y113 may promote the attested membrane trafficking function of PGRMC1. To stimulate further experimentation, we also discuss that heme-mediated dimerization of PGRMC1 and membrane trafficking may be mutually exclusive functions. These roles could potentially be reciprocally regulated by phosphorylation/dephosphorylation at Y113. It follows that the phosphorylation status of PGRMC1 should be further explored in order to better understand many of its proposed biological functions.

## MULTIFACETED PGRMC1 FUNCTION IS IMPLICATED IN DISEASE

**Figure 1:**
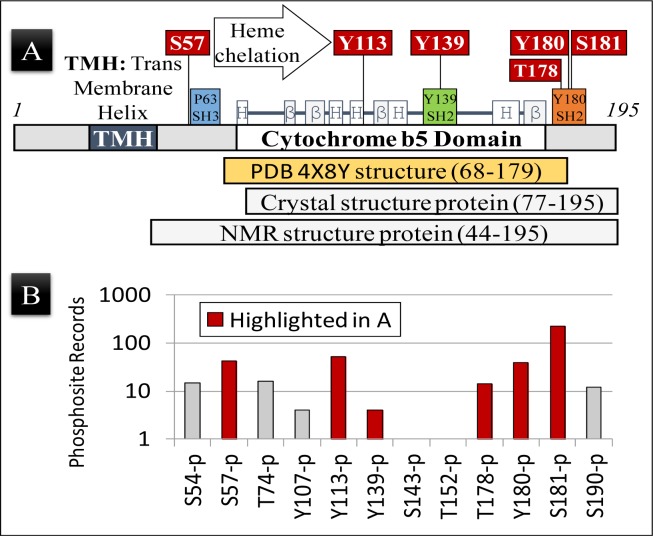
PGRMC1 is phosphorylated on key regulatory amino acid residues **A.** Schematic representation of the PGRMC1 protein showing the position of secondary structural elements common to PGRMC1 and related cytochrome *b*_5_ fold proteins [1]. The positions of highlighted regulatory phosphorylations observed in (B) below are indicated, as are the amino acids included in the NMR structure deposited as PDB 4X8Y, and the amino acid residues used to obtain crystal and NMR structures [4]. Not all regulatory residues lie within the region of the determined structure. **B.** The number of records for each specific PGRMC1 phosphopeptide identified by high throughput mass spectrometry, as documented in the Phosphosite Database (www.phosphosite.org) for Uniprot entry O00264 (PGRMC1, human). S, T, and Y are amino acids according to the standard one-letter code and numbers are the O00264/PGRMC1 amino acid residue number. Note that the observed phosphopeptide frequency could be related to both the efficiency of peptide ionization/detection in mass spectrometry as well as to the biological frequency of occurrence.

Progesterone receptor membrane component 1 (PGRMC1) is 195 residue membrane-bound protein which contains a short luminal peptide, a single *N*-terminal transmembrane domain, and a *C*-terminal cytochrome *b*_5_-related heme-binding domain (Figure 1A). It is thought to be involved in cancers of the female reproductive tract and disorders of the central nervous system (*e.g.*, Alzheimer's disease). Moreover, PGRMC1 has been attributed with controlling diverse functions including neuronal guidance, membrane trafficking, modulation of cytochrome P450 activity, progesterone-responsiveness, steroidogenesis and regulation of the mevalonate pathway, *etc.* [1–3]. To be involved in such disparate functions the activity of the PGRMC1 protein is probably intricately regulated.

## MODIFICATION OF PGRMC1 SIGNALING BY PHOSPHORYLATION

The recent structure of PGRMC1 published in *Nature Communications* [4] confirmed many predictions reached by modeling PGRMC1 to the known structures of related proteins [1, 5, 6]. Furthermore, exciting new advances in our knowledge of PGRMC1 function have now emerged [4]. Most importantly, heme-binding dimerizes the protein, which is required for its interaction with the cytochrome P450 enzyme that is responsible for the hydroxylation and inactivation of the anti-cancer chemotherapeutic, doxorubicin [4].

The new structure [4] also illuminates previously unrecognized insights into the potential regulation of PGRMC1 function by phosphorylation. The apparent perplexing diversity of PGRMC1 function may be related to predicted binding sites for interacting SH2- or SH3-domain signaling proteins (Figure 1A) [1, 7]. These interactions have been predicted to be regulated by phosphorylation at specific sites within the PGRMC1 molecule [1, 8]. Briefly, consensus SH2-domain target sequences are centered on Y139 and Y180 (Figure 1A), and could require tyrosine phosphorylation for SH2-proteins to bind [1, 8]. This binding may activate events in a similar manner to the recruitment of proteins to tyrosine-phosphorylated hormone receptors [9]. In fact, access to both the SH3-target sequence of PGRMC1 centered on P63, and the SH2-target sequence centered on Y180 (Figure 1A), have been predicted to be inhibited by casein kinase 2 (CK2)-mediated phosphorylation of adjacent serines [8].

## PGRMC1 IS DIFFERENTIALLY PHOSPHORYLATED *IN VIVO*

In a proteomics study, PGRMC1 was observed to be differentially phosphorylated between estrogen receptor-positive or estrogen receptor-negative breast cancers [8]. However, the sites of differential phosphorylation were not determined [8]. As such, PGRMC1 was one of the first proteins whose early cancer association was not at the level of expression, but at the level of differential phosphorylation [8]. In addition, Munton *et al*. observed Y180-phosphorylated PGRMC1 to be more than ten-fold more abundant in post-synaptic density fractions of primary mouse neurons than in synaptic membranes [10].

Considering the combination of differential phosphorylation and the presence of signaling signatures in PGRMC1, one of us predicted that PGRMC1 occupies a signaling nexus position in a PGRMC1 signaling system [8] with properties resembling a network hub protein [11]. As such, alternative states of PGRMC1 phosphorylation may exert higher level pleiotropic effects that could regulate critical functions in normal and pathological cell biology [8].

## KEY PREDICTED PGRMC1 REGULATORY RESIDUES ARE PHOSPHORYLATED *IN VIVO*

Phosphorylation of Y180 is predicted to be sterically inhibited by phosphorylation of S181 by CK2, and the observed T178 phosphorylation (Figure 1A) should also affect protein interactions with Y180. Phosphorylation of S57 by CK2 could also putatively sterically attenuate binding of SH3-domains to the SH3-target sequence centered at P63 [1, 7].

Notably, CK2 is a constitutive kinase responsible for 20% of the mammalian phosphoproteome [12]. Hence, S57 and S181 are expected to be constitutively phosphorylated under most conditions, potentially negatively regulating PGRMC1 interactions with SH2- and SH3-domain-containing proteins. Both S57 and S181 are among the most commonly observed phosphopeptides in the Phosphosite Database [13], along with Y113 and Y180 (Figure 1B). Finally, and significantly, heme chelation *via* a tyrosinate ion at Y113 would probably be impossible if Y113 were phosphorylated [4]. Significantly, we report here that these phosphorylation events all occur *in vivo* (Figure 1B).

## TYROSINE PHOSPHORYLATION AND SH2-BINDING TO Y180 MAY ALLOSTERICALLY REGULATE PGRMC1 SIGNALING

**Figure 2:**
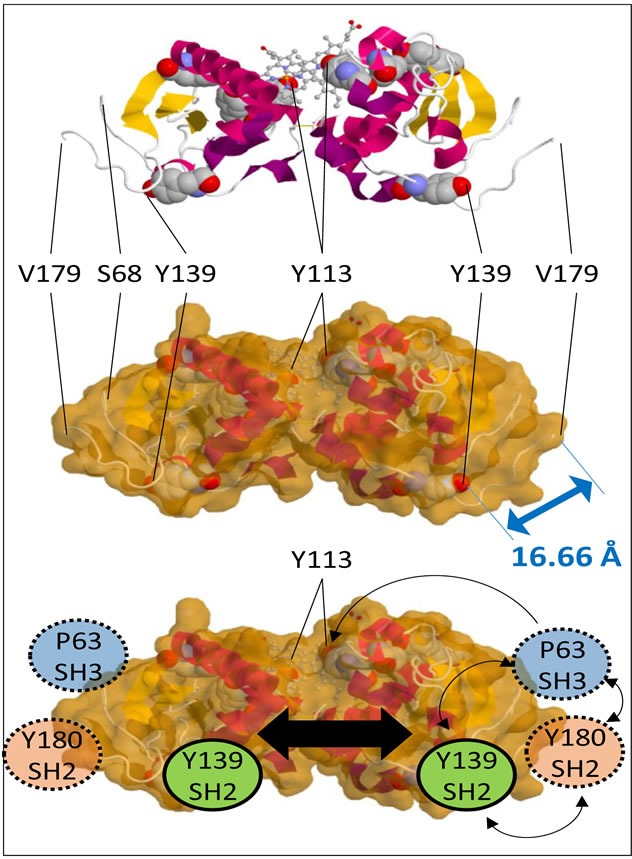
PGRMC1 contains a putative tripartite signaling platform Top: Schematic representation of the PGRMC1 dimer with tyrosines depicted in space filling models and heme in a ball and stick format. Middle: Solvent accessible surface retaining space filling tyrosines. The double-headed arrow indicates a distance of 16.66 Å between the Y139 hydroxyl group to the alpha carbon of V179. Bottom: Schematic depiction of PGRMC1 showing adjacent protein interaction motifs on the opposite surface to bound heme, consisting of an SH3-target sequence centred on P63, and SH2-target sequences centred on Y139 and Y180. These residues could co-localize and activate signaling proteins upon occupation. Dimerization could enhance this effect (large black arrow). Images were made with the JSmol Viewer that is available at http://www.rcsb.org/pdb/explore/jmol.do?structureId=4X8Y.

In the SH2-target sequence centered at Y139, the structure of PGRMC1 now reveals that while the Y139 hydroxyl is exposed [4], the aromatic ring is partially buried by residues immediately C-terminal to the conserved cytochrome *b*_5_ domain (Figure 2). This fact was not apparent from previous structural modeling [1, 5]. Phosphorylation of Y139 by a tyrosine kinase probably requires a prior allosteric structural change to expose the aromatic ring. In addition, binding by an SH2-domain to Y139 would probably require the phosphorylated tyrosine to be solvent-exposed.

Considering this latter proposal, we suggest that binding of an SH2-domain protein to Y180, or an SH3-domain protein to the SH3-target sequence centered at P63, may produce conformational changes that expose Y139 and/or T178. Relevant to this, the structure of the polypeptide backbone surrounding Y180 was unstable under both crystallization and NMR conditions [4]. This may indicate that the Y180 SH2-target sequence and the adjacent S181 CK site are available to interact with enzymes *in vivo*. Tyrosine phosphorylation of Y180 and recruitment of an SH2-domain protein may induce allosteric structural changes to the surrounding PGRMC1 conformation.

Notably, from the Y139-OH group to C-alpha of V179, the atomic distance is only 16.66Å (Figure 2), in a region of PGRMC1 that may be conformationally flexible. Thus, recruitment of an SH2-domain protein to Y180 could conformationally alter the PGRMC1 structure around Y139. This allosteric hypothesis is consistent with the observation that the side chain hydroxyl of T178 is also oriented towards the protein interior in the published structure [4], although it could be phosphorylated (Figure 1B).

## THE SH3-TARGET SEQUENCE AT P63 IS EXPOSED TO SOLVENT

The PGRMC1 structure from residues 68-179 was obtained by NMR from a protein containing PGRMC1 amino acids 44-195 [4] (Figure 1A). Like the potential SH2-target at Y180, the conformation of the putative SH3-target sequence centered at P63 was also disordered in solution, as judged by NMR [4]. This observation suggested that the SH3-target sequence is also in a flexible non-structured region of PGRMC1. This deduction is made with the caveat that interactions with the missing membrane could potentially stabilize tertiary structural elements.

An SH3-domain protein bound to the P63 SH3-target sequence would be adjacent to SH2-domain proteins bound to both Y139 and Y180 (Figure 2). This tripartite binding platform for different signaling proteins is located on the opposite surface of both the monomer and dimer from the ligand-binding pocket, as was previously suspected [1]. The simultaneous recruitment of enzymes and their substrates to this signaling platform should activate signal transduction processes similar in principle, if not identical, to the activations associated with classical tyrosine-phosphorylation of membrane receptors [9].

## HEME-BINDING AND VESICLE TRAFFICKING MAY BE MUTUALLY EXCLUSIVE - THE ROLE OF Y113

Phosphorylation of Y113 could cause steric interference incompatible with heme-binding [4], and yet Y113 phosphorylation is observed more frequently than that of any residue except S181 (Figure 1B). Runko and Kaprielian [14] noted sequence conservation in PGRMC1 of several potential YXX(Φ) consensus motifs (where Φ represents aliphatic amino acids), which are also called immunoreceptor tyrosine-based activation motifs (ITAMs). When tyrosine is phosphorylated at the end of a helix, ITAMs are involved in functions such as internalization of receptors on the cytoplasmic membrane after ligand binding [14] (reviewed in Ref. [1]). Considering this, from the NMR structure of PGRMC1 [4], Y113 represents the sole ITAM motif positioned at the end of a helix in PGRMC1.

Since PGRMC1 modulates vesicle trafficking [2, 15–18], it can be suggested that phospho-Y113 could be involved, and that heme-binding and vesicle trafficking by PGRMC1 may represent mutually exclusive functions. Lack of heme-binding could expose a hydrophobic surface patch, and this may be involved in new molecular interactions required for processes such as membrane trafficking (or other interactions). This could suggest that at least two important cellular functions of PGRMC1 are reciprocally regulated by Y113 phosphorylation.

## IS HEME-BINDING REQUIRED FOR EGFR INTERACTION?

Kabe *et al*. [4] concluded that heme-mediated dimerization of PGRMC1 was required for association with epidermal growth factor receptor (EGFR) since Y113 was required for both heme chelation and EGFR interaction. Furthermore, they observed no phosphorylation of Y113. However, it remains possible that transient Y113 phosphorylation is required for membrane trafficking to co-localize PGRMC1 and EGFR, which unfortunately their experimental design cannot exclude [4]. Intriguingly, EGFR association with PGRMC1 has been shown to reside topologically either in a lumenal compartment, or in a highly protease-resistant cytoplasmic protein complex [16], such as that which may exist at the membrane of a coated vesicle.

## PERSPECTIVE

While these predictions and suggestions above remain to be experimentally substantiated, all of the described phosphorylations occur *in vivo* (Figure 1B), and the mutation of both S57 and S181 dramatically change PGRMC1 function in MCF7 cells [8]. To achieve tyrosine phosphorylation of PGRMC1, first a phosphatase may need to dephosphorylate S57 and/or S181. Recruitment of an Abl-like kinase to the P63-centered SH3-target sequence [1] could potentially result in phosphorylation of tyrosines. The relationship or dependence of phosphorylation or heme-binding to the presence of progesterone also remains unclear.

In summary, considering our analysis, we suggest that future research should focus on characterizing the state of PGRMC1 phosphorylation and its involvement in important disease processes such as cancer [8, 19] and Alzheimer's disease [18]. Furthermore, the identification and functional characterization of kinases and phosphatases involved in these events will be crucial to our understanding of the PGRMC1 signaling network.

## References

[1] Cahill MA (2007). Progesterone receptor membrane component 1: an integrative review. J Steroid Biochem Mol Biol.

[2] Ahmed IS, Chamberlain C, Craven RJ (2012). S2R (Pgrmc1): the cytochrome-related sigma-2 receptor that regulates lipid and drug metabolism and hormone signaling. Expert Opin Drug Metab Toxicol.

[3] Losel RM, Besong D, Peluso JJ, Wehling M (2008). Progesterone receptor membrane component 1—many tasks for a versatile protein. Steroids.

[4] Kabe Y, Nakane T, Koike I, Yamamoto T, Sugiura Y, Harada E, Sugase K, Shimamura T, Ohmura M, Muraoka K, Yamamoto A, Uchida T, Iwata S (2016). Haem-dependent dimerization of PGRMC1/Sigma-2 receptor facilitates cancer proliferation and chemoresistance. Nature Commun.

[5] Rohe HJ, Ahmed IS, Twist KE, Craven RJ (2009). PGRMC1 (progesterone receptor membrane component 1): a targetable protein with multiple functions in steroid signaling, P450 activation and drug binding. Pharmacol Ther.

[6] Mifsud W, Bateman A (2002). Membrane-bound progesterone receptors contain a cytochrome b5-like ligand-binding domain. Genome Biol.

[7] Peluso JJ, Pappalardo A, Losel R, Wehling M (2006). Progesterone membrane receptor component 1 expression in the immature rat ovary and its role in mediating progesterone's antiapoptotic action. Endocrinology.

[8] Neubauer H, Clare SE, Wozny W, Schwall GP, Poznanovic S, Stegmann W, Vogel U, Sotlar K, Wallwiener D, Kurek R, Fehm T, Cahill MA (2008). Breast cancer proteomics reveals correlation between estrogen receptor status and differential phosphorylation of PGRMC1. Breast Cancer Res.

[9] Ostman A, Hellberg C, Bohmer FD (2006). Protein-tyrosine phosphatases and cancer. Nature reviews Cancer.

[10] Munton RP, Tweedie-Cullen R, Livingstone-Zatchej M, Weinandy F, Waidelich M, Longo D, Gehrig P, Potthast F, Rutishauser D, Gerrits B, Panse C, Schlapbach R, Mansuy IM (2007). Qualitative and quantitative analyses of protein phosphorylation in naive and stimulated mouse synaptosomal preparations. Mol. Cell. Proteomics.

[11] Goh KI, Cusick ME, Valle D, Childs B, Vidal M, Barabasi AL (2007). The human disease network. PNAS USA.

[12] Meggio F, Pinna LA (2003). One-thousand-and-one substrates of protein kinase CK2?. FASEB J.

[13] Hornbeck PV, Chabra I, Kornhauser JM, Skrzypek E, Zhang B (2004). PhosphoSite: A bioinformatics resource dedicated to physiological protein phosphorylation. Proteomics.

[14] Runko E, Kaprielian Z (2004). Caenorhabditis elegans VEM-1, a novel membrane protein, regulates the guidance of ventral nerve cord-associated axons. J. Neuroscience.

[15] Hampton KK, Craven RJ (2014). Pathways driving the endocytosis of mutant and wild-type EGFR in cancer. Oncoscience.

[16] Ahmed IS, Rohe HJ, Twist KE, Craven RJ (2010). Pgrmc1 (progesterone receptor membrane component 1) associates with epidermal growth factor receptor and regulates erlotinib sensitivity. J Biol Chem.

[17] Mir SU, Jin L, Craven RJ (2012). Neutrophil gelatinase-associated lipocalin (NGAL) expression is dependent on the tumor-associated sigma-2 receptor S2RPgrmc1. J Biol Chem.

[18] Izzo NJ, Xu J, Zeng C, Kirk MJ, Mozzoni K, Silky C, Rehak C, Yurko R, Look G, Rishton G, Safferstein H, Cruchaga C, Goate A (2014). Alzheimer's Therapeutics Targeting Amyloid Beta 1-42 Oligomers II: Sigma-2/PGRMC1 Receptors Mediate Abeta 42 Oligomer Binding and Synaptotoxicity. PLoSOne.

[19] Ahmed IS, Rohe HJ, Twist KE, Mattingly MN, Craven RJ (2010). Progesterone receptor membrane component 1 (Pgrmc1): a heme-1 domain protein that promotes tumorigenesis and is inhibited by a small molecule. J Pharmacol Exp Ther.

